# Broaden sources and reduce expenditure: Tumor-specific transformable oxidative stress nanoamplifier enabling economized photodynamic therapy for reinforced oxidation therapy

**DOI:** 10.7150/thno.49731

**Published:** 2020-08-21

**Authors:** Xiaoyu Xu, Binyao Huang, Zishan Zeng, Jie Chen, Zeqian Huang, Zilin Guan, Meixu Chen, Yanjuan Huang, Chunshun Zhao

**Affiliations:** School of Pharmaceutical Sciences, Sun Yat-sen University, Guangzhou, 510006, P. R. China.

**Keywords:** Oxidative stress, oxidation therapy, reactive oxygen species, economized photodynamic therapy, broaden sources and reduce expenditure

## Abstract

Cancer cells immersed in inherent oxidative stress are more vulnerable to exogenous oxidative damages than normal cells. Reactive oxygen species (ROS)-mediated oxidation therapy preferentially aggravating tumor oxidative stress to disrupt redox homeostasis, has emerged as an effective and specific anticancer treatment. Herein, following an ingenious strategy of “broaden sources and reduce expenditure”, we designed a versatile tumor-specific oxidative stress nanoamplifier enabling economized photodynamic therapy (PDT), to achieve synergistic oxidative stress explosion for superior oxidation therapy.

**Methods:** Cinnamaldehyde (CA) as a therapeutic ROS generator was first conjugated to hyaluronic acid (HA) through acid-labile hydrazone bond to synthesize tailored amphiphilic HA@CA conjugates, which could surprisingly self-assemble into uniform nanofibers in aqueous media. Photosensitizer protoporphyrin (PpIX) was efficiently encapsulated into HA@CA nanofibers and transformed HA@CA nanofibers to final spherical HA@CAP.

**Results:** With beneficial pH-responsiveness and morphology transformation, improved bioavailability and selective tumor accumulation, HA@CAP combining ROS-based dual chemo/photodynamic treatment modalities could induce cytotoxic ROS generation in a two-pronged approach to amplify tumor oxidative stress, termed “broaden sources”. Moreover, utilizing CA-induced H_2_O_2_ production and cascaded Fenton reaction in mitochondria to consume intracellular overloaded Fe(II), HA@CAP could skillfully block endogenic heme biosynthesis pathway on site to restrain undesired elimination of PpIX for economized PDT, termed “reduce expenditure”. Both *in vitro* and *in vivo* results demonstrated the superior antitumor performance of HA@CAP.

**Conclusion:** This study offered an inspiring strategy of “broaden sources and reduce expenditure” to specifically boost tumor oxidative stress for reinforced oxidation therapy.

## Introduction

Reactive oxygen species (ROS), including hydrogen peroxide (H_2_O_2_), superoxide radical (O_2_^•-^), hydroxyl radical (•OH), and singlet oxygen (^1^O_2_), play vital roles in cell signaling and homeostasis in biological processes 1, 2. Compared with normal cells, cancer cells immersed in intrinsic oxidative stress are more vulnerable to further oxidative damages induced by exogenous ROS 3-5. Therefore, oxidation therapy aggravating oxidative stress to an extent beyond the threshold of cancer cells, selectively disrupting tumor redox homeostasis without affecting normal cells, has emerged as an effective strategy for tumor-specific treatment 6, 7.

Current ROS-mediated oxidation therapy involves photodynamic therapy (PDT), chemotherapy, sonodynamic therapy, radiotherapy and chemodynamic therapy (CDT). PDT mainly relies on the ^1^O_2_ produced by activated photosensitizers with oxygen under light irradiation to exterminate tumor cells and tissues 8-10. Protoporphyrin (PpIX) is a commonly used photosensitizer in clinical PDT. It can be endogenously formed from bioprecursor 5-aminolevulinic acid (ALA) via cellular heme biosynthesis pathway 11, 12. However, the lacking tumor selectivity and poor water solubility of PpIX, seriously restrict its clinical application. Great efforts have been devoted to solving these issues and various versatile nanocarriers such as polymeric nanoparticles 13, 14, micelles 15, 16, liposomes 17, peptide-based nanoparticles 18, 19 and inorganic nanoparticles 20, have been fabricated to enhance photosensitivity and delivery efficiency of PpIX for improved PDT. However, these delivery strategies mainly emphasize how to increase the cellular internalization of administrated PpIX for enhanced intracellular ROS generation, while the subsequent intracellular elimination of PpIX seems to be overlooked.

Following the endogenic heme biosynthesis pathway, PpIX as a naturally arising precursor will be further metabolized into light inactive heme in mitochondria through ferrous ions Fe(II) insertion, which is catalyzed by ferrochelatase 21. As cancer cells intrinsically require more iron to maintain aberrant metabolism and cell proliferation, tumor tissues have excessive iron than normal tissues 22-24. Therefore, in addition to the common shortcomings as other photosensitizers, this exclusive and inherent metabolism inside iron overload cancer cells can also induce undesired and rapid PpIX elimination to considerably compromise PDT efficacy 25. Typically, in clinical ALA-induced PDT, intracellular available PpIX accumulation depends not only on the amount of ALA entering cancer cells, but also on the inactivation of PpIX caused by Fe(II) insertion under catalysis of ferrochelatase 26, 27. Except for increasing the given dose of ALA, scavenging intracellular labile Fe(II) pool using iron chelators such as EDTA and desferioxamine (DFO) to inhibit the intracellular metabolic conversion of PpIX to ineffective heme, has been verified as an effective strategy to increase PpIX accumulation for enhanced PDT efficacy 28, 29.

Cinnamaldehyde (CA) containing an active Michael acceptor pharmacophore has been widely exploited as a potential chemotherapeutic agent for tumor-specific oxidation therapy 30-33. CA can promote intracellular ROS generation mainly in mitochondria and amplify oxidative stress to induce ROS-mediated mitochondrial dysfunction and caspase activation for cancer cells apoptosis 34, 35. Furthermore, CA has been confirmed to effectively elevate intracellular H_2_O_2_ level, and induce massive •OH generation through Fenton reaction for enhancing routine CDT efficacy 36, 37. CDT is an emerging anticancer treatment utilizing iron-mediated Fenton reactions to convert intracellular mild H_2_O_2_ into highly cytotoxic •OH for cellular damage 38, 39. In intracellular Fe(II)-mediated Fenton reaction systems, Fe(II) decomposes H_2_O_2_ to generate •OH with itself oxidized into Fe(III), while the regeneration of Fe(II) from Fe(III) is very slow 40, 41.

Inspired by the common mechanism inducing ROS overproduction to destroy redox homeostasis for cell death, we assume that the combination of CA-mediated chemotherapy with PpIX-mediated PDT could be a rational two-pronged broaden sources strategy to realize synergistic oxidative stress explosion for amplified oxidation therapy. Particularly, as a therapeutic redox regulator, CA can initiate intracellular oxygen and light irradiation-independent ROS generation and cascaded Fenton reaction to aggravate tumor oxidative stress, effectively offsetting the limitations of hypoxic tumor microenvironment, light attenuation and potential photo-toxicity in routine PDT 42-46. Meanwhile, CA-initiated Fenton reaction could skillfully decrease intracellular Fe(II), restraining undesired PpIX biotransformation in mitochondria for economized PDT, termed “reduce expenditure”. However, with poor bioavailability caused by oxidable instability of aldehyde group and deficient tumor tissues specificity, the clinical application of CA still remains a challenge.

**Scheme 1:**
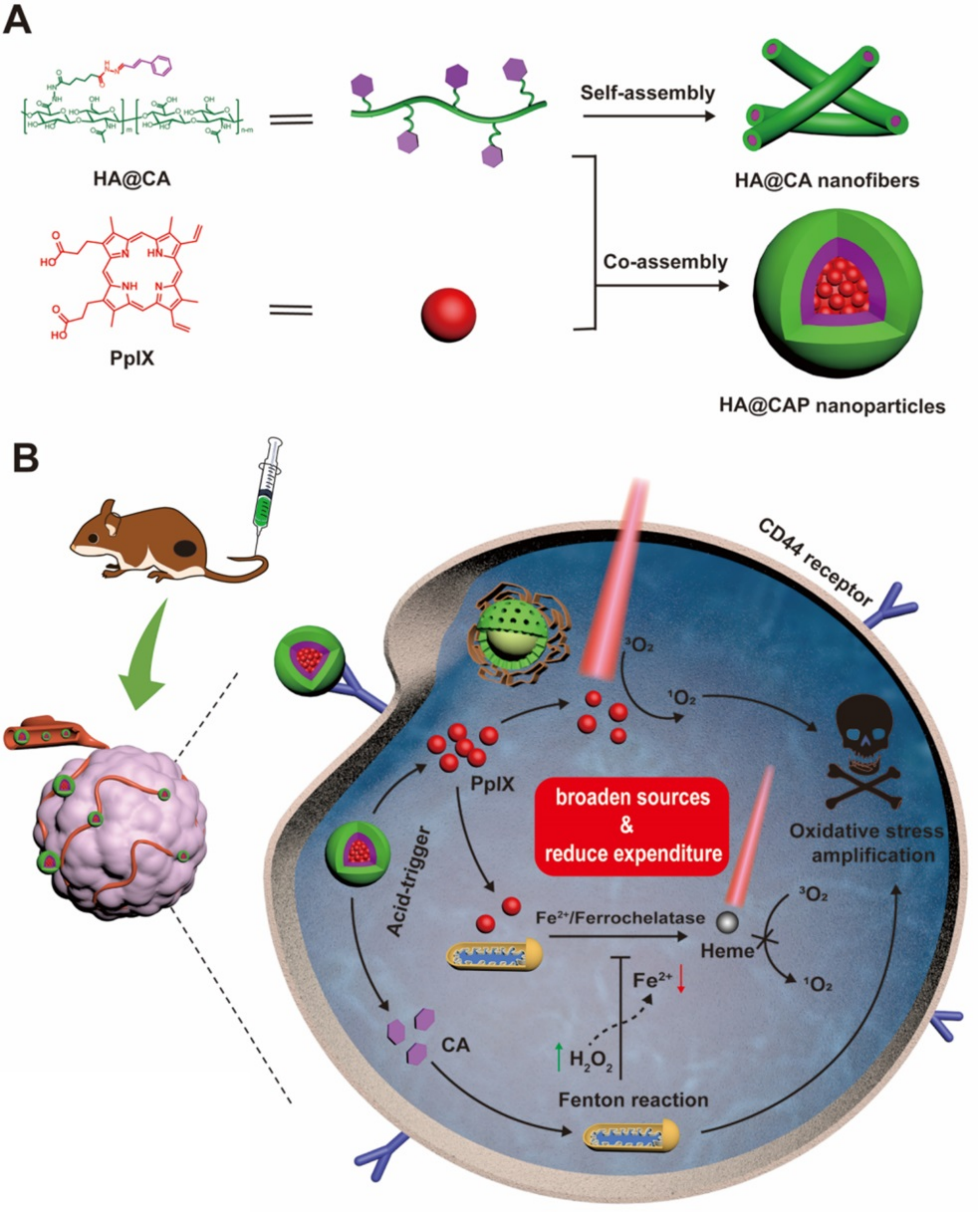
Schematic illustration of tumor-specific transformable oxidative stress nanoamplifier enabling economized photodynamic therapy for reinforced oxidation therapy. (**A**) Schematic illustration of the self-assembly process of HA@CA nanofibers and HA@CAP nanoparticles. (**B**) Schematic illustration of *in vivo* therapeutic mechanism of HA@CAP for reinforced oxidation therapy with an ingenious strategy of “broaden sources and reduce expenditure”.

Keeping all these issues in mind, we designed an integrated tumor-specific oxidative stress nanoamplifier enabling economized PDT, to achieve superior ROS-mediated oxidation therapy with an ingenious strategy of “broaden sources and reduce expenditure”. Hyaluronic acid (HA), a natural biocompatible polysaccharide, was employed as a tumor-targeted vehicle owing to the strong affinity for specific CD44 receptors overexpressed on surface of cancer cells 47. As illustrated in **Scheme 1A**, to protect vulnerable aldehyde group for improved stability and tumor tissues specificity, lipophilic CA was conjugated to hydrophilic HA through acid-labile hydrazone bond to synthesize amphiphilic HA@CA conjugates. HA@CA could spontaneously self-assemble into nanofibers with efficient drug loading in aqueous media. Then, hydrophobic photosensitizer PpIX with a macrocyclic conjugated structure was efficiently encapsulated into HA@CA nanofibers via π-π stacking and hydrophobic interactions to obtain HA@CAP. Interestingly, by adjusting the hydrophobicity of self-assembly, the incorporation of PpIX could transform the morphology of previous self-assembled HA@CA from nanofibers to beneficial spherical nanoparticles.

As depicted in **Scheme 1B**, owing to passive enhanced permeability and retention (EPR) effect and active CD44 receptor-mediated endocytosis, HA@CAP nanoparticles could preferentially accumulate in tumor sites after intravenous injection. Through the cleavage of hydrazone bond under intracellular acidic lysosome environments, HA@CAP would disassemble and release the pre-protected CA with encapsulated PpIX. The released PpIX could be activated to produce ^1^O_2_ for tumor PDT upon laser irradiation, while CA could induce intracellular oxygen and light irradiation-independent ROS including H_2_O_2_ generation in mitochondria and initiate iron-mediated Fenton reaction, thus aggravating intracellular oxidative stress together for enhanced cells killing. Meanwhile, the intracellular Fe(II) consumption attributed to CA-initiated Fenton reaction could skillfully inhibit undesired PpIX elimination on site, eventually realizing simultaneous enhanced PpIX internalization and suppressed PpIX clearance in cancer cells for economized and reinforced PDT. Both *in vitro* and *in vivo* results validated that HA@CAP could achieve superior antitumor efficacy through combining CA-mediated chemotherapy with PpIX-mediated PDT. This study provided a promising strategy of “broaden sources and reduce expenditure” to aggravate tumor oxidative stress for reinforced ROS-mediated oxidation therapy.

## Materials and Methods

### Materials, cell culture and animals

HA (Mw = 10 kDa) was obtained from Shandong Freda Biochem Co., Ltd. (Shandong, China). CA, adipic acid dihydrazide (ADH), 1-ethyl-3-(3-(dimethylamino)propyl) carbodiimide hydrochloride (EDC), 9,10-anthracenediyl-bis(methylene)dimalonic acid (ABDA) and fluorescein diacetate (FDA) were purchased from Aladdin Reagent Inc. (Shanghai, China). PpIX was obtained from Howei Pharm (Guangzhou, China). Propidium iodide (PI), 2′,7′-dichlorofluorescein diacetate (DCFH-DA), 4',6-diamidino-2-phenylindole (DAPI) and 3-(4,5-dimethylthiazol-2-yl)-2,5-diphenyl tetrazolium bromide (MTT) were obtained from Sigma-Aldrich (St. Louis, USA). BES-H_2_O_2_-Ac was purchased from Wako chemical (Tokyo, Japan). 3′,6′-bis (diethylamino)-2-(4-oxopent-2-en-2-ylamino) spiro (isoindoline-1,9′-xanthen)-3-one was obtained from Heliosense Biotechnology Inc. (Xiamen, China). Hydroxyphenyl fluorescein (HPF) was purchased from Shanghai Maokang Bio. Co. (Shanghai, China). The mouse melanoma cells B16F10 and NIH-3T3 cells were purchased from the Laboratory Animal Center of Sun Yat-sen University. The B16F10 cells were cultured in RPMI 1640 medium containing 10% fetal bovine serum (FBS) and 1% penicillin-streptomycin at 37 °C in a humidified 5% CO_2_ incubator. Female C57BL/6 mice (18-22 g) were provided by the Laboratory Animal Center of Sun Yat-sen University (Guangzhou, China). All experimental procedures were approved and supervised by the Institutional Animal Care and Use Committee of Sun Yat-sen University.

### Synthesis and characterization of HA@CA conjugates

HA was first modified with ADH to introduce massive hydrazide groups. In brief, 3 g of HA and 6 g of ADH were mixed in 100 mL of distilled water and stirred for 30 min. The pH of reaction mixture was regulated to 4.7, and then 3 g of EDC were added to the above solution to initiate reaction. The pH of mixture was maintained at 4.7 by adding 0.1 M HCl solution. The final reaction mixture was stirred overnight and purified by dialysis against distilled water (Mw 1 kDa) for 48 h to remove excess reactants and byproducts, finally obtain HA-ADH through lyophilization. Then, HA@CA conjugates were achieved via the formation of hydrazone bonds between hydrazide groups of HA-ADH and aldehyde groups of CA. Briefly, 2 g of HA-ADH and various amounts of CA at feed ratio of 3%, 5%, 8%, 10% and 15% were dissolved in 100 mL of distilled water with two drops of acetic acid added and stirred overnight. The mixture was purified by sequential dialysis against 10% ethanol/water and water (Mw 1 kDa) to remove any unreacted CA, and finally lyophilized to obtain HA@CA. The chemical structures of HA-ADH and HA@CA were determined by ^1^H NMR spectra (Bruker AvanveIII 400MHz) and Fourier transform infrared (FT-IR) spectra (EQUINOX 55, Bruker). The grafting rate of HA@CA was confirmed by a UV-vis spectrophotometer (UV2600; Techcomp) according to the calibration curve of CA in 10% DMSO/H_2_O solution.

### Preparation and characterization of HA@CA nanofibers

For HA@CA nanofibers preparation, 5 mg of lyophilized powder of HA@CA was dispersed in 5 mL of DMSO, and then added dropwise into 10 mL of deionized water with vigorous stirring. Next, the mixture was dialyzed against distilled water for 24 h to remove DMSO, and finally filtered to obtain assembled HA@CA nanofibers solution. To determinate the critical micelle concentration (CMC) of amphiphilic HA@CA for self-assembly, the pyrene fluorescence probe method was employed. HA@CA synthesized with feed ratio at 10% were prepared at various concentrations (3~75 mg/L), to which the pyrene in acetone was added to a predetermined concentration of 1.2×10^-4^ mg/mL. After vigorous ultrasonication for 30 min, the fluorescence excitation spectra of pyrene-loaded HA@CA were measured by a fluorescence spectrometer (HORIBA, Fluoromax-4, USA) (λem = 390 nm). The ratio (I_338_/I_333_) of excitation intensity at 338 nm and 333 nm was plotted against the logarithm of HA@CA concentrations, the concentration at the inflection point was CMC.

### Preparation and characterization of HA@CAP

Self-assembled HA@CAP was prepared by co-assembly of HA@CA and PpIX in aqueous media. Briefly, 0.1 g of PpIX in DMSO was dropwise added into 50 mL of distilled water containing 1 g of HA@CA and stirred for 2 days in the dark. To further remove any unpackaged PpIX and DMSO, the mixture was purified by sequential dialysis against 10% ethanol/water and water (Mw 1 kDa), and then filtered by 0.45 μM Millipore filters to gain final HA@CAP nanoparticles. Size distribution and zeta potentials of HA@CA and HA@CAP were measured by a Malvern Zetasizer instrument (Zetasizer Nano ZS90, Malvern, UK). Their morphologies were observed by a transmission electron microscope (TEM, JEM-1400, JEOL, Japan). The content of PpIX in HA@CAP was determined using fluorescence spectrophotometer. The UV-vis and fluorescence spectra of HA@CAP dissolved in aqueous solution and free PpIX dissolved in water or DMSO were obtained by UV-vis spectroscopy and fluorescence spectroscopy, respectively.

### *In vitro* measurement of ^1^O_2_ production upon NIR light irradiation

Using ABDA as a water-soluble indicator, the ^1^O_2_ generation from free PpIX and HA@CAP upon NIR light irradiation in aqueous solution was evaluated. Free PpIX and HA@CAP with equivalent dose of PpIX were dissolve in ABDA working solution (0.205 mg/mL) at a final concentration of PpIX (6.4 μg/mL). Then, the mixture was intermittently irradiated with an infrared laser (630 nm, 500 mW/cm^2^) (Changchun New Industries Optoelectronics Technology, China) at an interval of 5 min. At predetermined time points, the solution was thoroughly mixed for UV-vis spectra scanning ranging from 220 nm to 450 nm. The ABDA degradation rate is calculated by the absorbance of ABDA decreased at 401 nm.

### *In vitro* drug release

The acid-triggered release of CA from HA@CA was firstly qualitatively confirmed by recording the shifts in absorption peak of UV-vis absorption spectra after acid treatment. To further assess pH-responsive CA release profiles, 5 mg of HA@CAP in 2 mL of distilled water were added into dialysis bags (Mw 1kDa), which were severally immersed in 50 mL of PBS (pH 5.0, 6.5 and 7.4). At appropriate time intervals, 1 mL of solution was taken out and the equal amount of fresh PBS was replenished. The concentration of CA in solution was measured using high performance liquid chromatography (HPLC) at 290 nm.

### Cellular uptake of HA@CAP

Confocal laser scanning microscope (CLSM) was utilized to detect the cellular uptake and distribution of HA@CAP in B16F10 cells. B16F10 cells were seeded on glass base dish in 12-well plates at a density of 1×10^5^ cells per well, and incubated overnight, then treated with fresh medium containing free PpIX, mixture of free PpIX with CA, HA@CAP (3 μM of free PpIX equivalents) separately for 4 h under the same conditions. Meanwhile, to verify the CD44 receptor-mediated endocytosis of HA@CAP with a competitive inhibition study, B16F10 cells were pretreated with 5 mg/mL of free HA for 1 h. After that, the culture medium was removed and rinsed twice with PBS, fixed with 4% paraformaldehyde for 15 min and stained with DAPI. Finally, the fixed cells were observed by CLSM with the excitation at 405 nm for DAPI and excitation at 630 nm for PpIX.

### Intracellular accumulation enhancement of PpIX

B16F10 cells were seeded in 96-well plates (5×10^3^ cells/well) overnight, then incubated with different formulations at same concentration of PpIX (3 μM) for 12 h. The formulations were prepared in serum-free medium by mixing PpIX with free CA, DFO, H_2_O_2_, FeCl_2_ and HA@CA at various concentrations (0 ~ 80 μM), respectively. The cell culture medium with PpIX alone was set as control group. Then, cells were washed twice with PBS and replaced with 200 μL of DMSO, followed by measuring the fluorescence intensity of PpIX in each well by a fluorescence spectrometer.

### Intracellular ROS generation

The intracellular ROS generated from various drug formulations were evaluated using DCFH-DA as a probe. Briefly, B16F10 cells were seeded onto 6-wells plates (2×10^5^ cells/well) and incubated for 24 h. Then, the culture medium was replaced with fresh culture medium containing various concentration of free CA, free PpIX, mixture of free PpIX with CA, HA@CA and HA@CAP (100 μM of free CA and 1 μM of free PpIX equivalents) for further 24 h of incubation at 37℃. The cells without any drug formulations treatments were used as control group. Afterward, the drug medium was replaced by medium containing DCFH-DA probe (10 μM) for another 30 min of incubation, and then washed three times with PBS. After that, cells were exposed to LED light irradiation (20 mW/cm^2^, 630 nm) for 10 min. Finally, the cells were harvest for flow cytometry measurements (Guava EasyCyte 6-2L, Merck Millpore). For CLSM imaging, after LED light irradiation, the cells were fixed with 4% polyformaldehyde and stained with DAPI. Finally, the cells were observed by CLSM.

### Intracellular H_2_O_2_ and •OH generation with Fe(II) consumption

To evaluate the H_2_O_2_ production capacity of CA in various formulations, B16F10 cells seeded on the glass coverslips in 12-wells plate at the density of 1×10^5^ cells per well, were incubated with CA (100 μM), HA@CA and HA@CAP for 12 h. Afterward, cells were stained with BES-H_2_O_2_-Ac (10 μM) for 30 min, followed by washed three times with PBS, fixed with 4% paraformaldehyde and stained with DAPI, then cells were observed using CLSM. For detecting intracellular •OH generation, treated B16F10 cells were rinsed and stained with specific •OH probe HPF (10 μM in PBS) for 60 min, followed by rinsed with PBS and imaged using CLSM. To further confirm the Fe(II) consumption, after treatment with H_2_O_2_ (positive control), CA and HA@CA for 12 h, the cells were stained with a ferric ion probe, 3′,6′-bis (diethylamino)-2-(4-oxopent-2-en-2-ylamino) spiro (isoindoline-1,9′-xanthen)-3-one (10 μM) at 37 °C for 30 min. Finally, the treated cells were rinsed with PBS and observed by CLSM.

### *In vitro* cytotoxicity and live/dead cell staining assay

The *in vitro* cytotoxicity of intermediate HA-ADH, CA and HA@CA against NIH-3T3 normal cells for 24 h was firstly determined by MTT assay. Briefly, 3T3 cells were seeded in 96-wells plates, followed by incubation at 37 °C under 5% CO_2_ for 24 h. Then, the cells were respectively treated with HA-ADH, CA and HA@CA at various concentrations. After incubation for 24 h, the cell viabilities were determined by MTT assay.

B16F10 cells seeded in 96-well plates were incubated with free CA, HA@CA, free PpIX and HA@CAP at different drug concentrations (50-600 μM of free CA equivalents, 0.5-2 μM of free PpIX equivalents), respectively. After 12 h of incubation, cells in LED light irradiation groups were treated with light irradiation (20 mW cm^-2^, 630 nm, 10 min) and cultured for further 12 h. Thereafter, 10 μL of MTT solution was added to each well for additional 4 h of incubation and then replaced with DMSO solution. The absorbance was measured at 490 nm using a microplate spectrophotometer (ELX800, Bio-Tek, USA).

Live/dead cell staining assay was carried out to visually assess the cytotoxicity. Briefly, B16F10 cells seeded in 24-well plates (5×10^4^ cells per well) were incubated with free CA, HA@CA, free PpIX and HA@CAP with or without irradiation at an equivalent concentration of CA (100 μM) or PpIX (1 μM). After incubation for 12 h, cells in LED light irradiation groups were treated with LED light irradiation (20 mW cm^-2^, 630 nm, 10 min) followed by additional 12 h of incubation. Then, cells were co-stained with FDA (5 μg/mL) and PI (5 μg/mL) for 10 min, washed three times with PBS, and finally observed by an inverted fluorescent microscope (IX73, Olympus, Japan).

### *In vivo* biodistribution of HA@CAP

B16F10 cells (1×10^6^) in 100 μL serum-free medium were subcutaneously inoculated to the right lower leg of C57BL/6 mice to develop tumor models. When tumor size reached 200∼300 mm^3^, the tumor-bearing C57BL/6 mice were randomly divided in two groups and intravenously injected with free PpIX and HA@CAP at an equivalent PpIX dose of 2.5 mg/kg. The mice were sacrificed at 0 h, 2 h, 4 h and 8 h postinjection. The tumor tissues and major organs (heart, live, spleen, lung and kidney) were isolated for *ex vivo* fluorescence imaging and intensity comparison using a small animal imaging system (Night OWL LB983, Berthold, Germany).

### *In vivo* antitumor efficacy

When the tumor size reached about 100-150 mm^3^, all tumor-bearing mice were randomly divided into seven groups (n=5) including saline group, saline with laser irradiation group, free CA group, HA@CA group, free PpIX with laser irradiation group, HA@CAP group and HA@CAP with laser irradiation group. All mice were intravenously treated with various formulations at day 1, 3 and 5 for a total of three times. After 4 h and 24 h postinjection, the mice in illuminated groups received laser irradiation (630 nm, 500 mW cm^-2^) for 10 min. The dosages of CA and PpIX were 5 mg/kg and 2.5 mg/kg, respectively. The tumor sizes and body weights of mice were recorded every other day, and the tumor volume was calculated using following formula: volume (mm^3^) = (tumor length) × (tumor width)^2^/2.

### Histological evaluation

All mice were sacrificed at the end of treatment. Tumor tissues in different groups were excised, fixed and further analyzed by hematoxylin and eosin (H&E) staining. Meanwhile, to assess potential systemic toxicity of various treatments, major organs including heart, liver, spleen, lung, and kidney at the end of antitumor experiment were collected for H&E staining histology analysis. The histological sections were observed under optical microscope (EVOS FL Auto, Life Technologies, USA).

### Statistical analysis

Data were presented as mean ± standard deviation (SD). Student's *t* test was performed to analyze the difference between different groups using SPSS Statistics 13.0 software. The obtained *p*-values (<0.05) were considered statistically significant.

## Results and Discussion

### Synthesis and characterization of HA@CA

**Figure 1:**
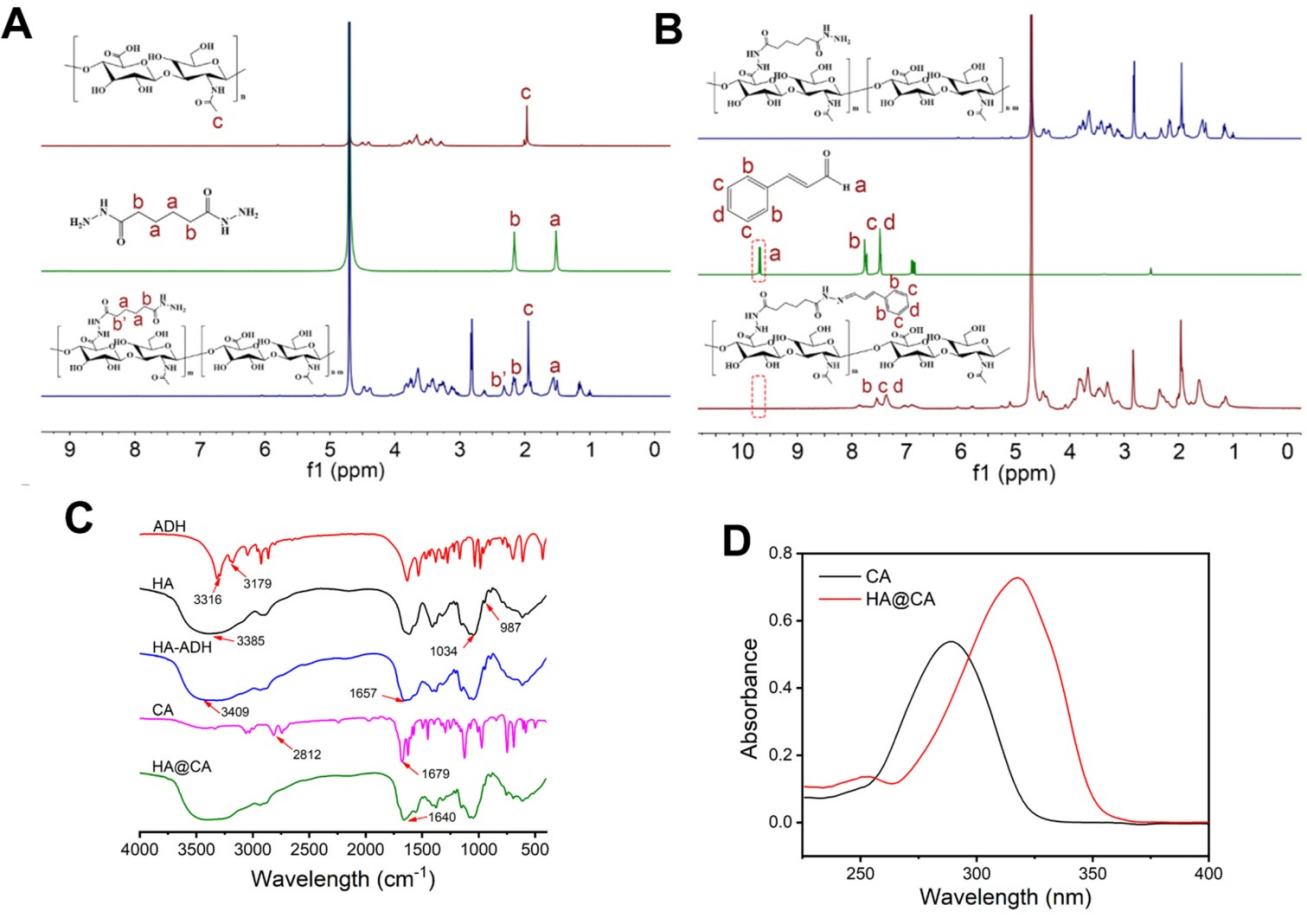
(**A**) The ^1^H NMR spectra of HA, ADH and HA-ADH in D_2_O. (**B**) ^1^H NMR spectra of HA-ADH in D_2_O, CA in DMSO-d_6_ and HA@CA in D_2_O. (**C**) FT-IR spectra of ADH, HA, HA-ADH, CA and HA@CA. (**D**) UV-vis absorption spectra of CA and HA@CA conjugate.

The synthetic routine of acid-sensitive HA@CA conjugates was shown in **Scheme S1**. First, to introduce massive hydrazide groups, ADH was chemically conjugated to HA under acid conditions to get HA-ADH. Then, through the formation of acid-labile hydrazone bonds between hydrazide groups of HA-ADH and aldehyde groups of CA, amphiphilic HA@CA conjugates were achieved. To substantiate the successful conjugation of ADH and CA, ^1^H NMR spectroscopy and FT-IR analysis were performed. As shown in **Figure 1A**, the characteristic peaks of ADH appeared at 2.160 ppm and 1.517 ppm were found at 2.322 and 1.465-1.655 ppm in the spectra of HA-ADH due to the amide condensation reaction with carboxyl groups of HA. Meanwhile, the successful conjugation of CA with HA-ADH was confirmed by the peaks of aromatic benzene at 6.691-7.689 ppm and the disappearance of aldehyde group peak in HA@CA spectra (**Figure 1B**). FT-IR results also verified the successful preparation of HA@CA. As illustrated in **Figure 1C**, there was a new peak at 1657 cm^-1^ in HA-ADH which was attributed to amide bond (C=O) formed from carboxyl groups of HA and hydrazine groups of ADH. Additionally, a newly appeared peak for imine stretching vibration (C=N) at 1640 cm^-1^ in the FT-IR spectrum of HA@CA indicated the presence of hydrazone bonds. Furthermore, the UV-vis absorption spectra of CA and HA@CA (**Figure 1D**) showed an obvious red shift of the maximum absorption from 290 nm for CA to around 310 nm for HA@CA, owing to the formation of hydrazone bond. Eventually, the determined loading content of CA in HA@CA prepared with different feed ratios was shown in **Table S1,** and HA@CA with a saturated CA content at 6.4%, indicating plentiful CA moieties in HA chains, was chosen for subsequent study.

### Preparation and characterization of HA@CA nanofibers

**Figure 2:**
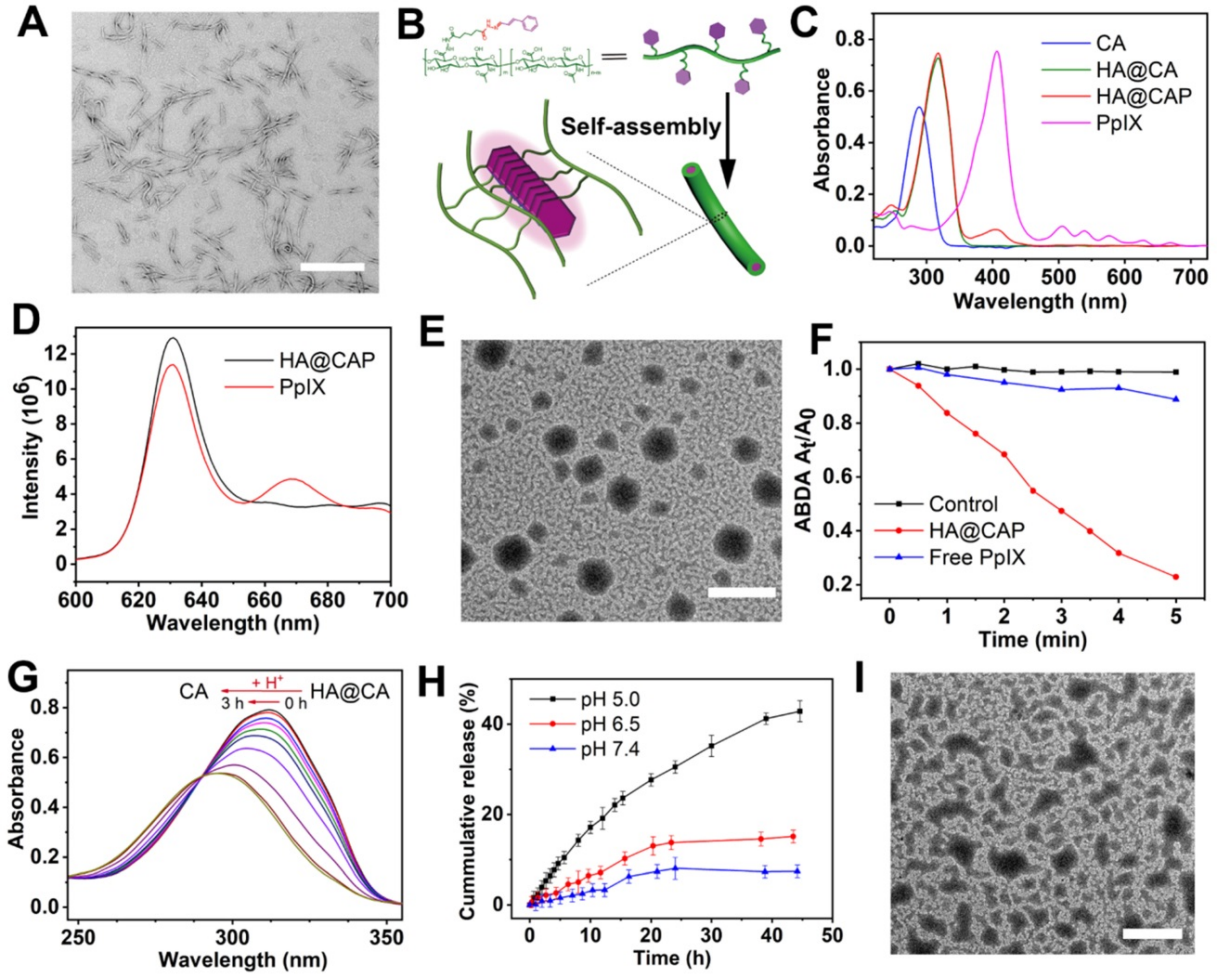
(**A**) TEM image of HA@CA nanofibers (scale bar: 500 nm). (**B**) Schematic illustration of the possible formation mechanism of HA@CA nanofibers in aqueous media. (**C**) UV-vis absorption spectra of CA, HA@CA, HA@CAP and PpIX. (**D**) Fluorescence emission spectrum of HA@CAP in H_2_O and PpIX in DMSO. (**E**) TEM image of HA@CAP nanopaticles (scale bar: 500 nm). (**F**) The extracellular ^1^O_2_ generation of HA@CAP and free PpIX in aqueous solution under laser irradiation. (**G**) The changes in UV-vis absorption spectra of HA@CA in acidic environment. (**H**) *In vitro* release of CA from HA@CAP under different pH conditions (7.4, 6.5 and 5.0). (**I**) TEM image of HA@CAP in PBS (pH 5.0). Scale bar: 500 nm.

Considering the amphiphilic structure of HA@CA conjugates, we expected that it could self-assemble into nanostructures in aqueous media. Surprisingly, amphiphilic HA@CA conjugate could further self-assemble into nanofibers in water. As shown in **Figure 2A**, HA@CA exhibited a uniform DNA-like double helix structure with a length of 300-400 nm and a width of 10-20 nm. Moreover, similar uniform nanofibers with helix structure were also observed in HA@CA conjugates prepared with various CA feed ratios, and the structure helicity of nanofibers increased gradually with the increase in grafting rate of CA (**Figure S1**). Similar to the possible mechanism reported in previous studies 48-50, we speculate that the driving force for nanofibers formation mainly comes from the powerful π-π stacking interactions between CA moieties in HA@CA conjugates. The massive planar aromatic benzene rings of CA in one or more chains have great tendency to overlap each other, forming one-dimensional nanofibers with a simulated “J-aggregate structures” through hydrophobic and π-π stacking interactions (**Figure 2B**). As an important parameter to evaluate the thermodynamic stability and self-assembly behavior of amphiphilic compound, the CMC of amphiphilic HA@CA was further measured as 22.77 μg/mL using a widely reported pyrene fluorescence probe method (**Figure S2**), indicating the favorable self-assembly capacity of HA@CA amphiphiles.

### Preparation and characterization of HA@CAP

PpIX was further encapsulated into the hydrophobic domain of HA@CA via hydrophobic interactions and π-π stacking to form final HA@CAP. The formation of HA@CAP was confirmed by UV-vis absorption spectra and fluorescence emission spectrum (**Figure 2C and 2D**). The characteristic absorption peak of PpIX at 405 nm was also detected in HA@CAP, and the fluorescent spectrum of HA@CAP showed same maximum emission at 630 nm with free PpIX. Interestingly, by adjusting the hydrophobicity of self-assembly, the PpIX loading unexpectedly modulated the morphology of self-assembled HA@CA nanostructures from nanofibers to spherical nanoparticles. As shown in **Figure 2E**, the TEM image of HA@CAP showed monodisperse, uniform spherical morphology. The mean particle size and zeta potential measured by dynamic light scattering (DLS) was about 220 nm (**Figure S3**) and negative charged at -23.3 mV (**Figure S4**), respectively. This notable transformation from nanofibers into nanoparticles could be ascribed to the destruction of previous hydrophilic/hydrophobic balance after hydrophobic PpIX loading. The tendency of π-π stacking between planar CA moieties to form one-dimensional nanofibers was disrupted by strong hydrophobic interactions between CA and PpIX 50, 51. The balance between HA@CA and PpIX tended to form spherical nanoparticles as the preferable lowest energy state, with HA as hydrophilic outer shell while CA and PpIX as hydrophobic inner core.

### The ^1^O_2_ production of HA@CAP under light irradiation

Photosensitizer PpIX can produce ^1^O_2_ under near infrared light irradiation for effective PDT. Therefore, it's necessary to verify the ^1^O_2_ production capacity of prepared HA@CAP. Water-soluble ABDA, which could react with ^1^O_2_ to induce the decrease of characteristic absorption at 401 nm, was used as an indicator. **Figure 2F** showed only less than 10% of ABDA was degraded during 5 min in free PpIX group, indicating inefficient ^1^O_2_ production caused by severe self-quenching effect of hydrophobic PpIX in aqueous solution. However, the HA@CAP group demonstrated a noticeable degradation of ABDA which was more than 80%, indicating that HA@CAP could effectively improve the dispersibility of PpIX and alleviate its aggregation-induced self-quenching in aqueous media. This result confirmed that HA@CAP could firmly ensure effective ^1^O_2_ generation under light irradiation, which showed promise for remarkable PDT efficacy.

### *In vitro* pH-responsiveness of HA@CAP

To elucidate the pH-sensitivity of HA@CAP, the release behavior of CA was monitored qualitatively and quantitatively. As shown in **Figure 2G**, the characteristic absorption of HA@CA at 310 nm was blue-shifted back to 290 nm after acid treatment, which was attributed to the generation of free CA in acidic condition. Furthermore, to mimic physiological release conditions, HA@CAP were placed in various pH (5.0, 6.5 and 7.4) to determine the content of released CA at different time points by HPLC. It could be seen from **Figure 2H** that only 7% of CA was released under normal physiological environment (pH 7.4) for 45 h, suggesting that HA@CAP was stable under physiological condition with negligible leakage. However, in acidic conditions (pH 6.5 or 5.0), the release of CA was significantly accelerated due to the acid-triggered cleavage of hydrazone bond. Particularly, the release of CA in pH 5.0 was 5.8 times more than that in normal physiological pH (pH 7.4). Moreover, the simultaneous acid-triggered size disassembly of HA@CAP was confirmed by TEM and DLS. In acidic conditions (pH 5.0), obvious structural disruption of previous nanoparticles morphology was observed by TEM (**Figure 2I**), and the average hydrodynamic diameter of completely disassembled HA@CAP could finally decrease to less than 10 nm (**Figure S5**). By contrast, the size of HA@CAP barely changed in PBS (pH 7.4) supplemented with 10 % FBS for 72 h, indicating good colloidal stability in physiological environment **(Figure S6)**. These results suggested that HA@CAP could keep stable during blood circulation to avoid premature leakage of CA, while disassemble under acidic endosomes/lysosomes environments to release CA quickly, which could contribute to enhancing the therapeutic effect of HA@CAP.

The acid-triggered size disassembly of HA@CAP was also confirmed by the fluorescence recovery phenomenon of PpIX. As shown in **Figure S7**, when HA@CAP was incubated under acidic environment, the fluorescence of PpIX increased rapidly with prolonged time. As was known to all, with π-π stacking and hydrophobic interactions, the fluorescence of PpIX was unavoidably quenched due to the aggregation of PpIX in hydrophobic cores of HA@CAP 52, 53. The structural collapse of HA@CAP could weaken the π-π stacking and hydrophobic interactions between PpIX and HA@CA, and reduce self-quenching of stacking PpIX in nanoparticles for fluorescence recovery. All above results evidenced beneficial pH-responsive release behavior and structural disassembly of HA@CAP, and these characteristics were expected to increase the drug delivery efficiency to specific acidic tumor microenvironment and reduce systemic side effects.

### Cellular uptake of HA@CAP

**Figure 3:**
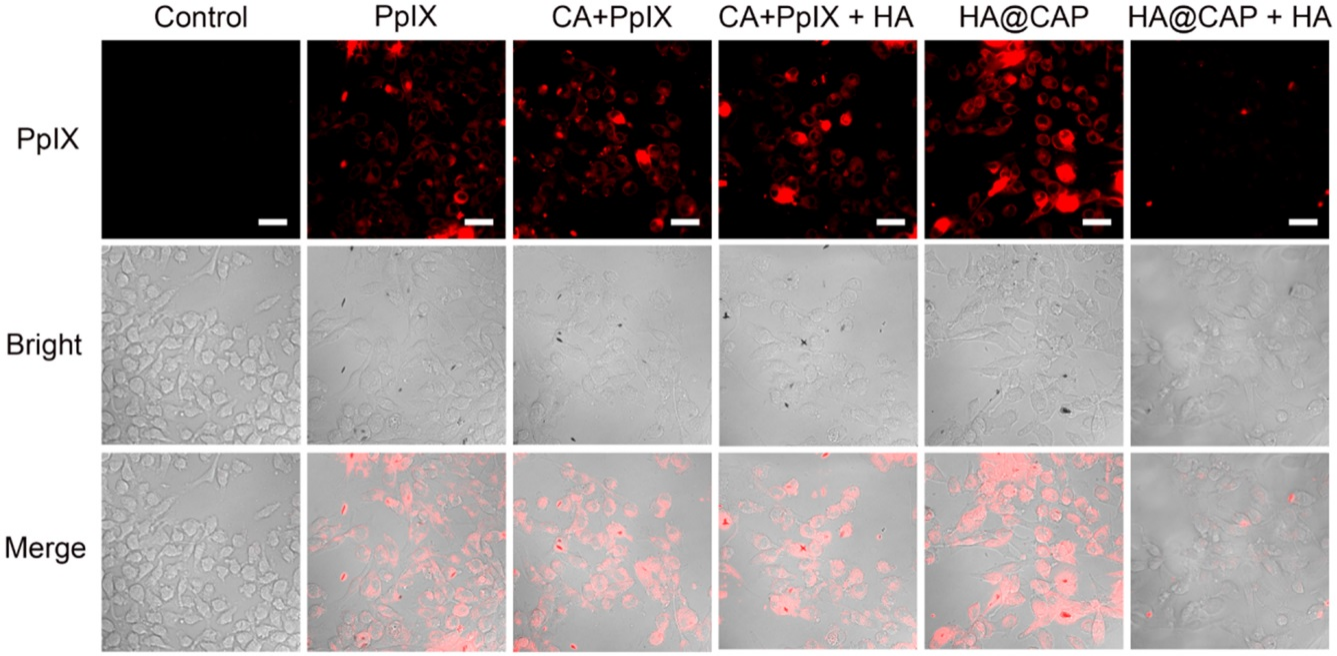
CLSM images of B16F10 cells incubated with PpIX, mixture of CA with PpIX, HA@CAP at equivalent PpIX concentration for 4 h. For competitive inhibition studies, B16F10 cells were precultured with 5 mg/mL of free HA for 1 h. Scale bar: 30 µm.

HA can actively target cancer cells via the specific binding with overexpressed CD44 receptors on the surface of cancer cells 47, thus the recognition of CD44 receptors contributes to the internalization of HA@CAP. As illustrated in **Figure 3**, after incubation with free PpIX or HA@CAP for 4 h, strong red fluorescence of PpIX was observed in both groups, suggesting efficient cellular uptake of lipophilic free PpIX molecule as well as HA@CAP nanoparticles. Compared with HA@CAP group, the fluorescence intensity was highly weaker in HA@CAP with free HA group, indicating their insufficient internalization into cells. This difference could be attributed to the competition between massive free HA with HA@CAP for CD44 receptors binding, which would hinder the CD44 receptor-mediated endocytosis of HA@CAP. On the contrary, no evident difference of red fluorescence intensity was observed between the CA+PpIX group and CA+PpIX+HA group, indicating the pretreatment of free HA hardly hinder the cellular uptake of hydrophobic free PpIX molecule, which could directly penetrate the cell membrane. Moreover, it is worth noting that, compared with free PpIX group, a slightly stronger fluorescence intensity of PpIX was observed when cells were incubated with the mixture of free CA and PpIX (CA+PpIX group), which indicated that CA might have potential to enhance intracellular PpIX accumulation. Therefore, HA@CAP could be an ideal candidate for tumor specific treatment, which could not only preferentially enter cancer cells by specific CD44-mediated endocytosis, but also potentially enhance the accumulation of internalized PpIX.

### Intracellular accumulation enhancement of PpIX

**Figure 4:**
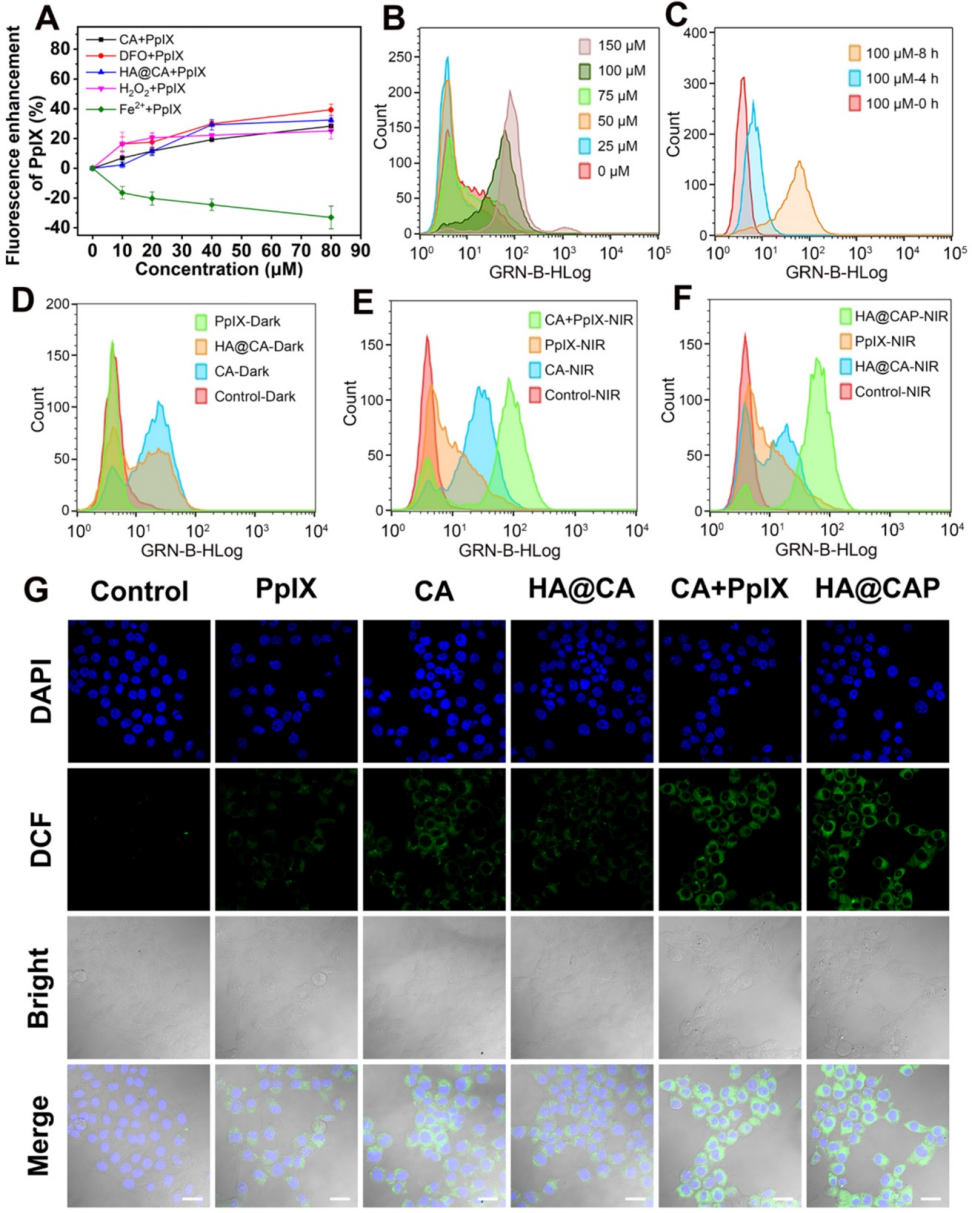
(**A**) Intracellular accumulation enhancement of PpIX induced by various drugs (CA, DFO, HA@CA, H_2_O_2_ and Fe^2+^) with different concentrations. (**B**) The intracellular ROS generation in B16F10 cells treated with various concentration of CA (0-150 µM) for 8 h, and (**C**) treated with 100 µM CA for different times (0, 4 and 8 h). (**D**) Flow cytometric analysis of intracellular ROS generation after different treatments under dark, and (**E, F**) with NIR laser irradiation. (**G**) CLSM observation of intracellular ROS generation after different treatments with NIR laser irradiation (Scale bar: 30 µm).

The adequate intracellular accumulation of photosensitizer is essential for effective PDT. As a direct precursor of heme in iron overload cancer cells, PpIX can be converted into light inactive heme via ferrochelatase-mediated insertion of Fe(II) into the porphyrin macrocycle. Therefore, reducing the intracellular content of Fe(II) to suppress intracellular metabolic conversion of PpIX is a good choice to enhance intracellular accumulation of PpIX. Using PpIX alone as control group, the effects of free CA, DFO, H_2_O_2_, FeCl_2_ and HA@CA on the intracellular accumulation of PpIX were investigated respectively. As expected, DFO, H_2_O_2_, CA and HA@CA exhibited considerable ability to enhance accumulation of PpIX inside cells with distinct enhancement of fluorescence intensities observed (**Figure 4A**). In contrast, the fluorescence intensity of PpIX in cells treated with FeCl_2_ gradually decreased with increasing Fe(II) incubated concentration. These experimental results clearly confirmed that exogenous Fe(II) supply could facilitate the conversion of PpIX to heme, while DFO, H_2_O_2_ could reduce the intracellular content of Fe(II) to suppress metabolic conversion of PpIX through iron chelation and Fenton reaction, respectively. Like DFO and H_2_O_2_, CA could also effectively enhance intracellular PpIX accumulation. The underlying mechanism might be that CA could induce the production of ROS including H_2_O_2_, thus also depleting the intracellular Fe(II) level for intracellular accumulation enhancement of PpIX. Therefore, integrated HA@CAP combining CA with PpIX could effectively reduce PpIX elimination for further economized and enhanced PDT.

### Intracellular ROS generation evaluation

Owing to the specific CD44-mediated cellular uptake and inhibited elimination of PpIX, HA@CAP has great promise to generate massive intracellular ROS for cancer cells killing. The intracellular ROS generation assay was performed using ROS Assay Kit DCFH-DA. Initially, the ROS generation ability of CA was investigated to confirm the potential of CA for elevating intracellular ROS. **Figure S8** showed the representative gating strategy employed to obtain the histograms from flow cytometry assays. As expected, free CA could gradually increase intracellular ROS level with prolonged incubation time and increasing concentration (**Figure 4B and 4C**). Meanwhile, HA@CA overall showed a weaker mean fluorescence intensity of DCF than free CA (**Figure 4D**). As some previous studies have reported that long sized nanofibers are not conducive to cellular uptake 51, 54, this difference might be ascribed to the limited cellular internalization of HA@CA and delayed intracellular release of CA for initiating ROS generation.

As illustrated in **Figure 4E**, a moderately enhanced DCF fluorescence implying PDT effect was observed in free PpIX with NIR laser irradiation group, while cells co-treated with free CA and PpIX mixture followed by laser irradiation (CA+PpIX-NIR) exhibited much higher mean fluorescence intensity, revealing the remarkably enhanced intracellular ROS generation by dual-pathway. Particularly, as shown in **Figure 4F**, under same light irradiation condition, HA@CAP group (HA@CAP-NIR) exhibited higher fluorescence intensity than monotherapy groups (PpIX-NIR or HA@CA-NIR groups), indicating the superior oxidative stress amplification resulting from the combination of CA-mediated chemotherapy with PpIX-mediated PDT. CLSM imaging was further employed to visually assess the intracellular ROS production. As illustrated in **Figure 4G**, upon light irradiation, both CA+PpIX group and HA@CAP group showed significantly stronger green fluorescence than any other groups, which was consistent with the results of flow cytometry (**Figure 4E and 4F**).

### Intracellular H_2_O_2_ and •OH generation with Fe(II) consumption

**Figure 5:**
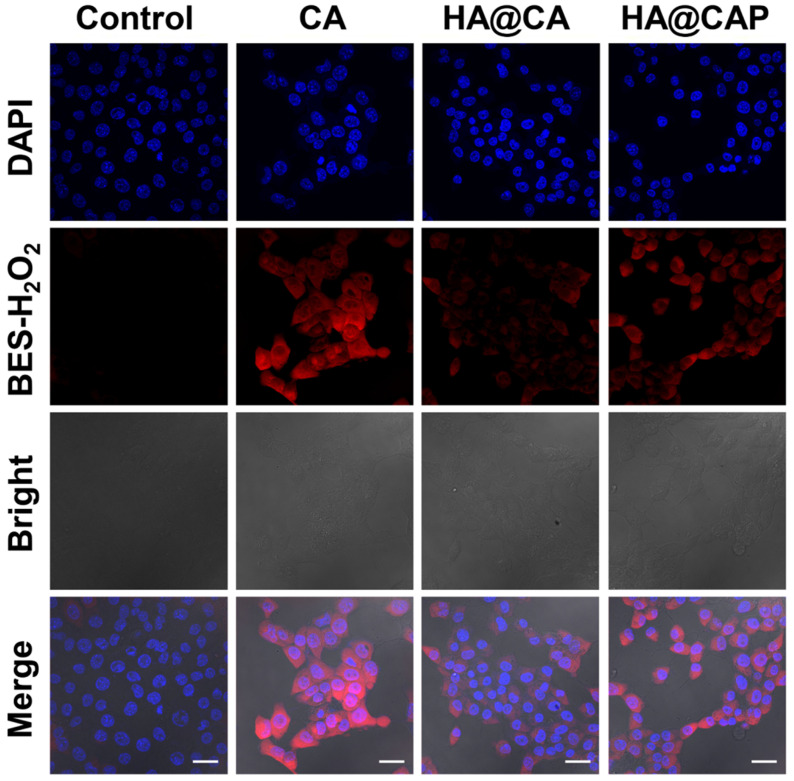
CLSM images of intracellular H_2_O_2_ generation in B16F10 cells after incubation with various formulations for 12 h using BES-H_2_O_2_-Ac as the specific H_2_O_2_ fluorescent probe. (Scale bar 30 µm).

To further confirm CA could really generate H_2_O_2_ to initiate Fenton reaction and suppress metabolic conversion of PpIX for enhanced intracellular accumulation, the H_2_O_2_ and •OH generation induced by CA was evaluated using a specific H_2_O_2_ probe BES-H_2_O_2_-Ac and •OH probe HPF, respectively. As illustrated in **Figure 5**, strong red BES-H_2_O_2_ fluorescence was distinctly observed in cells treated with free CA for 12 h. These results indicated that CA could generate massive intracellular H_2_O_2_, which could be utilized as the reactant to consume the intracellular content of Fe(II) through Fenton reaction for economized PpIX-mediated PDT. It is worth noting that, HA@CAP group showed a perceptible stronger BES-H_2_O_2_ fluorescence signal than HA@CA group under the same conditions. This H_2_O_2_ generation difference might be due to the nanostructure morphology dependent cellular uptake and spherical nanoparticles HA@CAP have higher cellular uptake efficiency than HA@CA nanofibers 51, 55. Therefore, this unique morphology transformation after incorporation of PpIX could be beneficial for preferable cellular uptake of HA@CAP.

**Figure 6:**
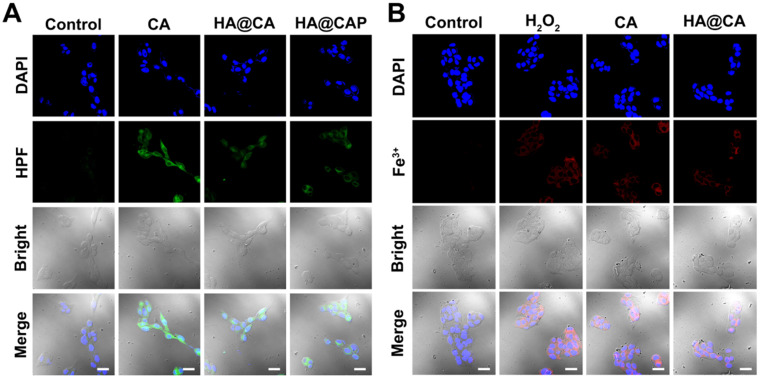
CLSM images of (**A**) intracellular •OH generation in B16F10 cells after incubation with various formulations for 12 h, (**B**) intracellular Fe(III) level of B16F10 cells after incubation with various formulations for 12 h. (Scale bar 30 µm).

Further, as expected, the cells treated with free CA, HA@CA or HA@CAP also showed observably stronger green fluorescence of HPF compared with control group, suggesting CA could indeed elevate intracellular H_2_O_2_ level and trigger the cascaded Fenton reaction inside iron overload cancer cells to produce •OH** (Figure 6A)**. To further prove the Fe(II) consumption during Fenton reaction, since there is no suitable specific Fe(II) probe, we temporarily used a commercial Fe(III) probe, 3′,6′-bis (diethylamino)-2-(4-oxopent-2-en-2-ylamino) spiro (isoindoline-1,9′-xanthen)-3-one, to detect the uprise of Fe(III) 39, 56. As shown in **Figure 6B**, similar to positive control group (H_2_O_2_), obvious red fluorescence could also be observed in the cells treated with free CA or HA@CA, suggesting the Fe(II)/Fe(III) conversion during CA-induced Fenton reaction, which contributed to economized PpIX-mediated PDT.

### *In vitro* cell cytotoxicity

**Figure 7:**
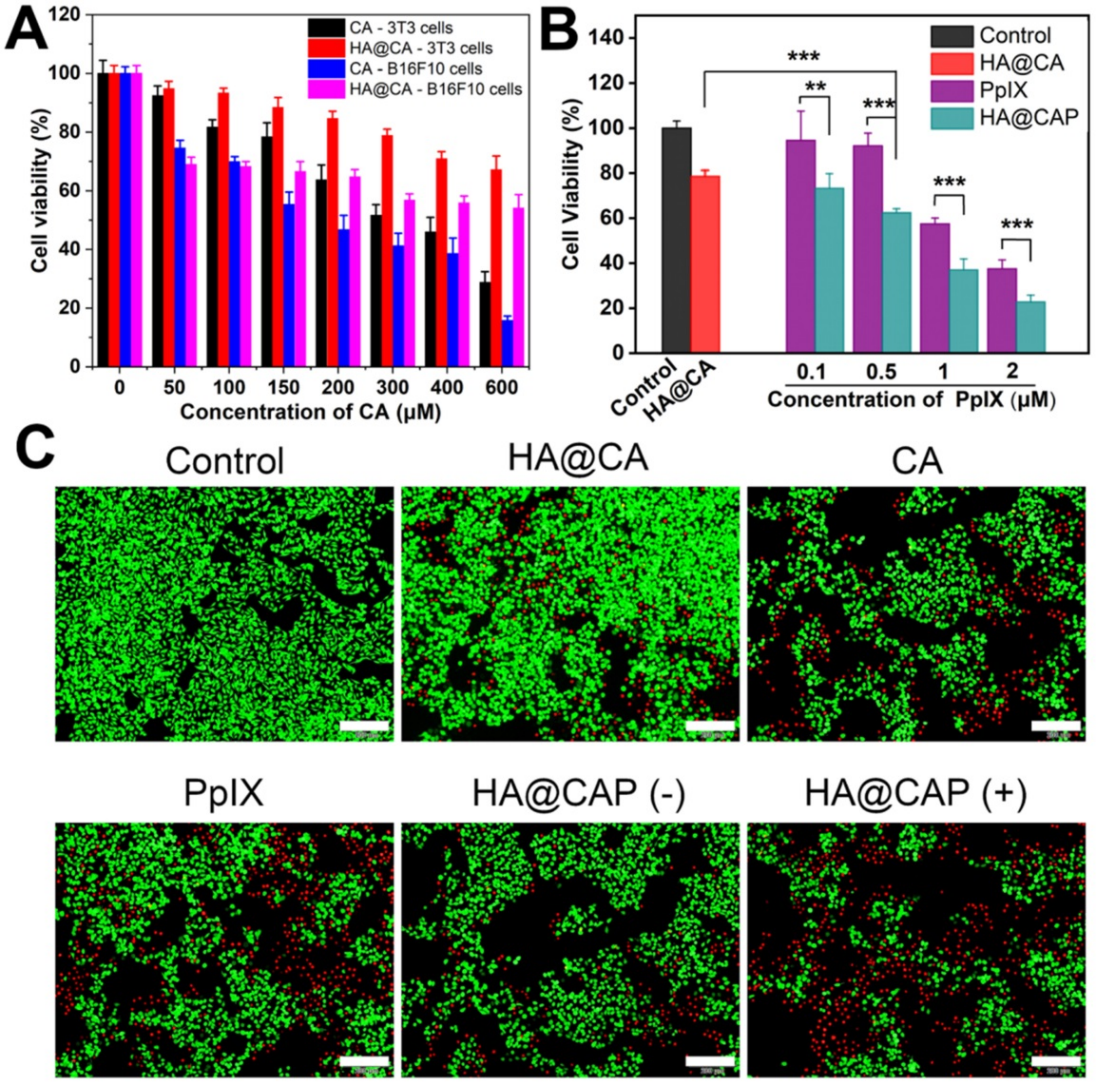
(**A**) Cell viability of NIH-3T3 cells and B16F10 cells after treated with various concentration of CA and HA@CA for 24 h. (**B**) Cell viability of B16F10 cells incubated with different formulations for 24 h. (**C**) Live/dead cell staining of B16F10 cells with different treatments. Scale bar = 200 µm.

First, the cytotoxicity of intermediate HA-ADH was evaluated using NIH-3T3 normal cells. As illustrated in **Figure S9**, the viabilities of NIH-3T3 cells remained above 95% after incubation with HA-ADH at the highest concentration (2 mg/mL), indicating the negligible cytotoxicity of biocompatible HA-ADH. Then, the cytotoxicity of free CA and HA@CA against NIH-3T3 cells and B16F10 cells were further evaluated. As shown in **Figure 7A**, for both NIH-3T3 cells and B16F10 cells, the cell viability decreased with the increasing concentrations of CA or HA@CA conjugate, suggesting a dose-dependent antiproliferative activity. Compared to lipophilic free CA, HA@CA showed lower cytotoxicity to both B16F10 cells and NIH-3T3 cells, which was probably attributed to the inadequate internalization and delayed acid-triggered time-consuming release of CA from HA@CA inside cells. Particularly, both CA and HA@CA showed a lower cytotoxicity to NIH-3T3 normal cells than B16F10 cancer cells at the equivalent concentrations of CA. The IC50 value of free CA at an incubation time of 24 h was 268.71 μM in B16F10 cancer cells while 373.29 μM in NIH-3T3 normal cells. These results suggested that their higher selectivity to inhibit cancer cells, which probably resulted from efficient cellular uptake toward cancer cells and further massive ROS generation for preferential destroy of tumor redox homeostasis.

In addition, B16F10 cells treated with free PpIX showed a dose-dependent phototoxicity upon light irradiation (**Figure 7B**), while LED light irradiation itself used in this study (20 mW cm^-2^, 630 nm) would not cause cells death (**Figure S10**). However, the cell cytotoxicity of HA@CAP, which combined CA-mediated chemotherapy with PpIX-mediated PDT, was markedly enhanced compared to either chemotherapy (HA@CA) or PDT alone (PpIX). Live/dead cell staining assay was further performed to visualize cell viability. FDA and PI were employed to stain live (green) and dead (red) cells, respectively. As displayed in **Figure 7C**, HA@CAP group with laser irradiation showed much larger area of red fluorescent dead cells compared to those of monotherapy groups, which was consistent with the results of MTT assays. Particularly, with the equivalent concentration of CA, HA@CAP without laser irradiation group showed an appreciable stronger red fluorescence signal than HA@CA group, suggesting the preferable anticancer effect of spherical nanoparticles HA@CAP resulting from the higher cellular uptake efficiency. This result was consistent with **Figure 5** and confirmed again the superiority of morphology transformation after incorporation of PpIX.

Above cellular results indicated that, HA@CAP combining ROS-based dual chemo/photodynamic treatment modalities, could realize the core concept of “broaden sources and reduce expenditure”. HA@CAP could effectively enter iron overload cancer cells and release the pre-protected CA with encapsulated PpIX, simultaneously induce massive ROS generation to aggravate cellular oxidative stress, as well as reduce intracellular PpIX elimination via CA-induced H_2_O_2_ production and cascaded Fenton reaction, eventually achieving synergistic oxidative stress amplification for superior oxidation therapy.

### *In vivo* biodistribution and tumor accumulation of HA@CAP

**Figure 8:**
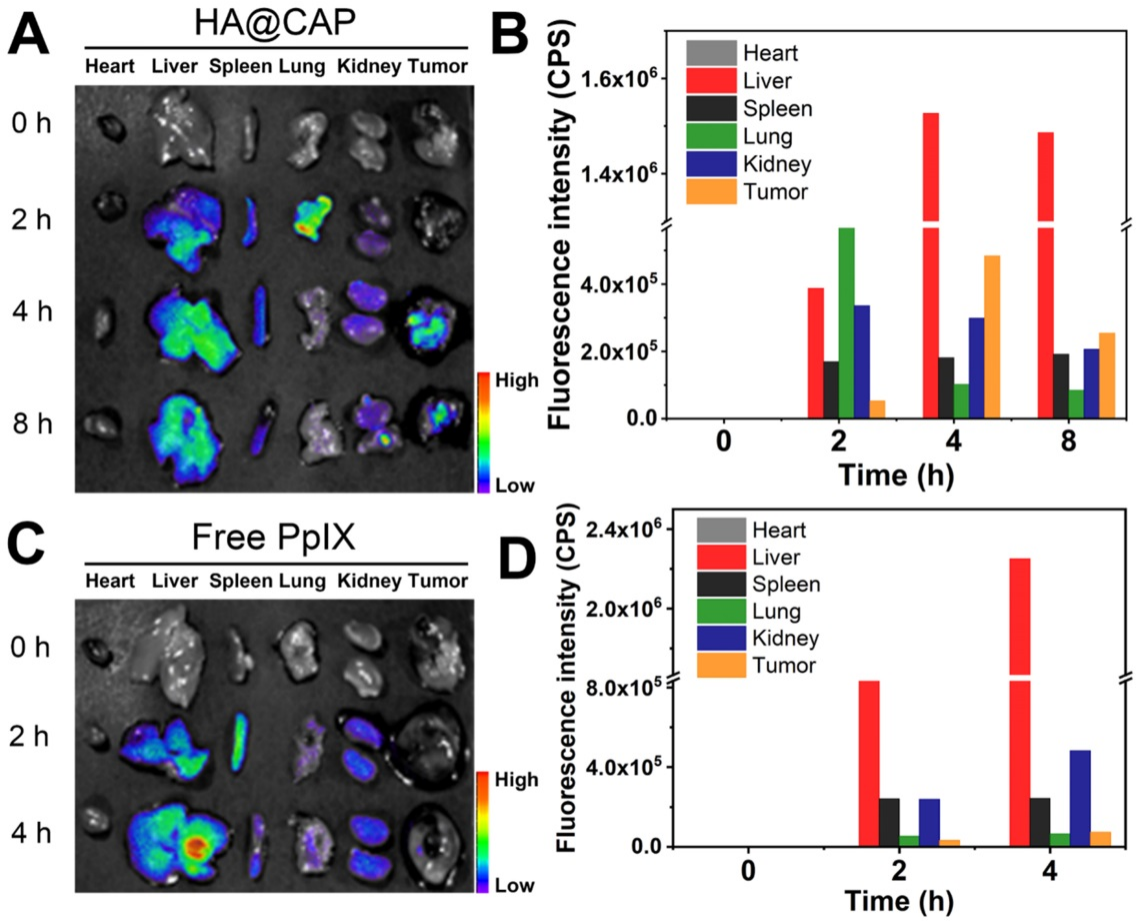
*In vivo* biodistribution and tumor targeting of HA@CAP in B16F10 tumor-bearing xenograft mice. (**A, C**) The *ex vivo* fluorescence imaging and (**B, D**) quantified mean fluorescence intensity values of excised major organs and tumors at different time points post-injection of HA@CAP and free PpIX.

The *in vivo* biodistribution of HA@CAP was examined using a small animal imaging system. Tumor-bearing C57BL/6 mice were intravenously injected with free PpIX and HA@CAP at an equivalent PpIX dose. The tumor tissues and major organs (heart, live, spleen, lung and kidney) were isolated at different time intervals for *ex vivo* fluorescence imaging (**Figure 8A and 8C**). As shown in **Figure 8C**, after injection with free PpIX, the fluorescence signal was mainly located in liver and kidney, indicating free PpIX exhibited nonspecific distribution. In contrast, HA@CAP maintained the certain intensity in liver during the whole observation period, and the tumor site showed gradually enhanced fluorescence with the maximum fluorescence appearing at 4 h post-injection (**Figure 8A**), suggesting improved tumor enrichment resulting from EPR effect for passive targeting and CD44 receptor recognition for active targeting. In addition, the semiquantitative result also verified the improved tumor tissue accumulation of HA@CAP (**Figure 8B and 8D**). In brief, the above results demonstrated that, HA@CAP could overcome common drawbacks of small molecule drugs, and efficiently accumulate at tumor sites with both passive targeting and active targeting ability, which was expected to improve *in vivo* therapeutic effect and reduce systemic toxicity.

### *In vivo* antitumor performance and biosafety of HA@CAP

**Figure 9:**
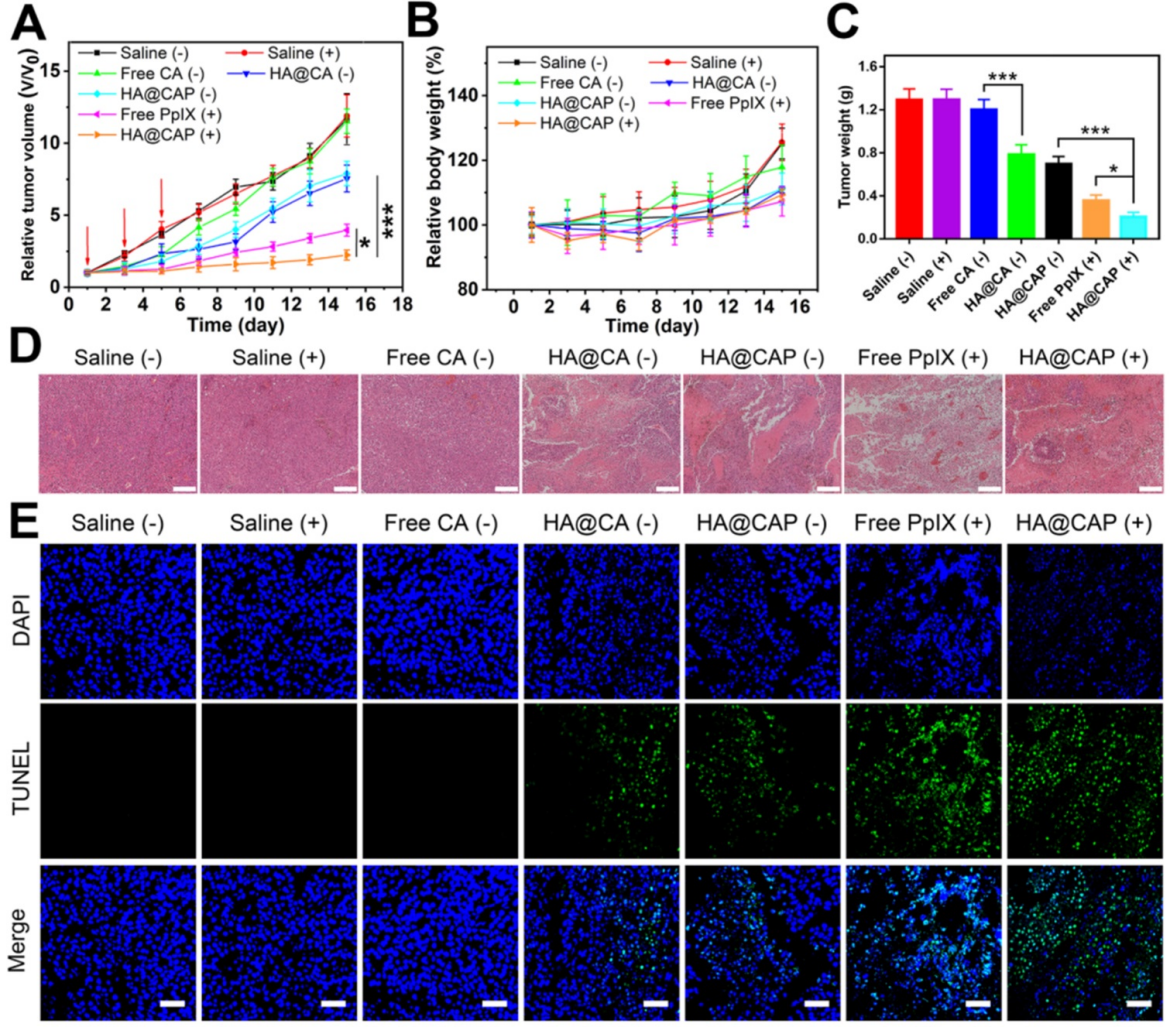
The *in vivo* antitumor performance of HA@CAP on the B16F10 tumor-bearing mice. Relative tumor volume growth curves (**A**) and body weight changes (**B**) of tumor-bearing mice after different treatments. (Red arrows indicated the time points of treatments). (**C**) Mean weight of excised tumor in various groups at the end of treatment. (**D**) H&E and (**E**) TUNEL staining of the tumor sections at the end of treatment. Scale bar: 200 µm (H&E); 50 µm (TUNEL). Data are represented as the mean ± SD (n = 5). *p < 0.05, **p < 0.01, and ***p < 0.001.

Inspired by the superior *in vitro* anticancer effect and improved *in vivo* tumor accumulation, the *in vivo* antitumor efficacy of HA@CAP was further evaluated using B16F10 tumor-bearing mouse model. As depicted in **Figure 9A**, compared with saline treatment groups with/without NIR irradiation, free CA group showed an unaffected tumor growth, and all tumor volumes at the end of treatment were about 12 times than initial tumors. Despite CA exhibited prominent cytotoxicity toward cancer cells *in vitro*, free CA could not inhibit *in vivo* tumor growth, which was ascribed to the extremely poor bioavailability of CA caused by rapid oxidation of aldehyde group and lack of tumor targeting ability. By contrast, both HA@CA group and HA@CAP without NIR irradiation group could effectively suppress tumor growth, suggesting that the self-assemblies of HA@CA conjugates could protect CA from adverse oxidation and afford beneficial tumor targeting ability for CD44 receptor, thus improving the biostability and tumor accumulation of CA for enhanced therapeutic effect. Meanwhile, free PpIX with NIR irradiation group showed remarkable tumor inhibition, indicating the effective PDT efficacy for epidermal melanoma after enough multiple-treatments. Notably, HA@CAP with NIR irradiation group exhibited the strongest tumor inhibition effect, associated with the smallest tumor volume (**Figure S11**) and weight (**Figure 9C**) in all treatment groups. This excellent antitumor performance confirmed the superior oxidation therapy by combining CA-mediated chemotherapy with PpIX-mediated PDT in integrated HA@CAP. Moreover, the H&E **(Figure 9D)** and TUNEL staining **(Figure 9E)** of tumor sections further confirmed that, HA@CAP with NIR irradiation group induced the most efficient tumor ablation with the most extensive tumor cell necrosis and apoptosis observed.

The biosafety evaluation was performed by monitoring the body weight changes during treatments and assessing the histological changes of major organs at the end of antitumor experiment. There was no obvious weight loss during various treatments, indicating the good biocompatibility of all treatments (**Figure 9B**). Meanwhile, no obvious pathological changes were observed between saline group and HA@CAP with laser irradiation group (**Figure S12**), which further indicated inappreciable system toxicity of HA@CAP. All these results demonstrated that HA@CAP as a tumor-targeted oxidative stress nanoamplifier combining CA-mediated chemotherapy with PpIX-mediated PDT, could realize superior antitumor efficacy with favorable biosafety.

## Conclusions

In summary, with an ingenious strategy of “broaden sources and reduce expenditure”, a tumor-specific transformable oxidative stress nanoamplifier for superior oxidation therapy was reported by combining CA-mediated chemotherapy with PpIX-mediated PDT. CA as a therapeutic redox regulator was conjugated to hydrophilic HA through acid-labile hydrazone bond to synthesize amphiphilic HA@CA conjugates. The pH-responsive HA@CA could realize the enhanced stability, beneficial tumor targeting ability, acid-triggered release of CA, and surprisingly self-assemble into nanofibers in aqueous media. Photosensitizer PpIX was efficiently encapsulated into HA@CA nanofibers and transformed the morphology of self-assembled HA@CA from nanofibers to preferable spherical nanoparticles for enhanced cellular uptake. With improved bioavailability and tumor accumulation, integrated HA@CAP induced massive cytotoxic ROS generation in dual-pathway for aggravating iron overload cancer oxidative stress, while blocked endogenic heme biosynthesis pathway to suppress the intracellular elimination of PpIX for economized PDT. This study provided an effective strategy of “broaden sources and reduce expenditure” to realize synergistic oxidative stress explosion for reinforced oxidation therapy.

## Supplementary Material

Supplementary figures and tables.

## Abbreviations

ROS: reactive oxygen species
H_2_O_2_: hydrogen peroxide
^1^O_2_: singlet oxygen
•OH: hydroxyl radical
PDT: photodynamic therapy
CA: cinnamaldehyde
HA: hyaluronic acid
PpIX: protoporphyrin Ⅸ
CDT: chemodynamic therapy
DFO: desferioxamine
EPR: enhanced permeability and retention
DMSO: dimethyl sulfoxide
EDC: 1-ethyl-3-(3-(dimethylamino)propyl) carbodiimide hydrochloride
ADH: adipic acid dihydrazide
FBS: fetal bovine serum
ABDA: 9,10-anthracenediylbis(methylene)dimalonic acid
CMC: critical micelle concentration
FDA: fluorescein diacetate
DCFH-DA: 2′,7′-dichlorofluorescein diacetate
HPF: hydroxyphenyl fluorescein
MTT: 3-(4.5-dimethyl-thiazol-2-yl)-2.5-diphenyl tetrazolium bromide
DAPI: 4',6-diamidino-2-phenylindole
PI: propidium iodide
H&E: hematoxylin and eosin
HPLC: high performance liquid chromatography
CLSM: confocal laser scanning microscope
TEM: transmission electron microscopy
DLS: dynamic light scattering
TUNEL: terminal deoxynucleotidyl transferase dUTP nick end labeling
