# Supplementary material for: Broaden sources and reduce expenditure: Tumor-specific transformable oxidative stress nanoamplifier enabling economized photodynamic therapy for reinforced oxidation therapy
Source: Theranostics. 2020 Aug 21;10(23):10513–30. doi: 10.7150/thno.49731 (PMC7482813; doi:10.7150/thno.49731)
Supplement: Supplementary file 1 — Supplementary figures and tables. [file thnov10p10513s1.pdf]

Supplementary material

Broaden sources and reduce expenditure: tumor-specific transformable oxidative stress nanoamplifier enabling economized photodynamic therapy for reinforced oxidation therapy

Xiaoyu Xu[1], Binyao Huang[1], Zishan Zeng, Jie Chen, Zeqian Huang, Zilin Guan, Meixu Chen, Yanjuan Huang, Chunshun Zhao*

School of Pharmaceutical Sciences, Sun Yat-sen University, Guangzhou, 510006, P. R. China.

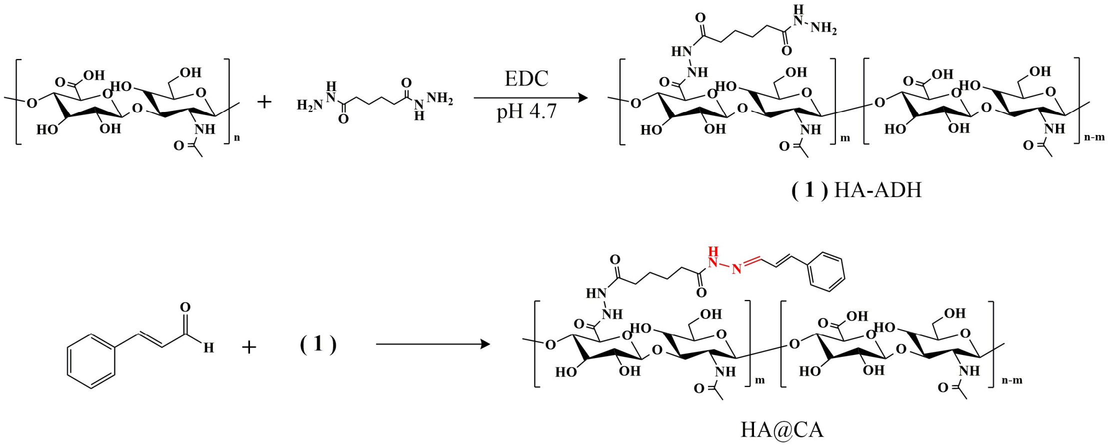

Scheme S1. The synthetic routes of HA@CA conjugates.

Table S1. The drug loading of CA in HA@CA under different CA feed ratio

	Feed ratio (m/m)	DL (m/m)
	3%	2.5%
	5%	4.8%
HA@CA	8%	5.4%
	10%	6.4%
	15%	6.14%

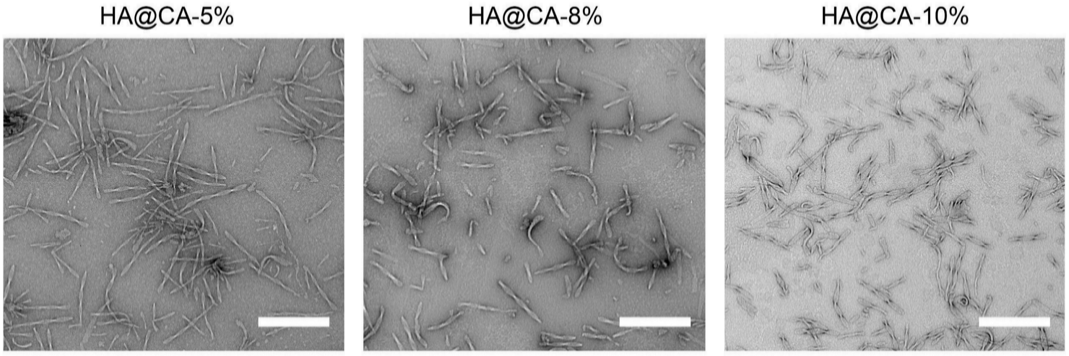

Figure S1. TEM images of HA@CA synthesized with different feeding rates of CA. Scale bar: 500 nm.

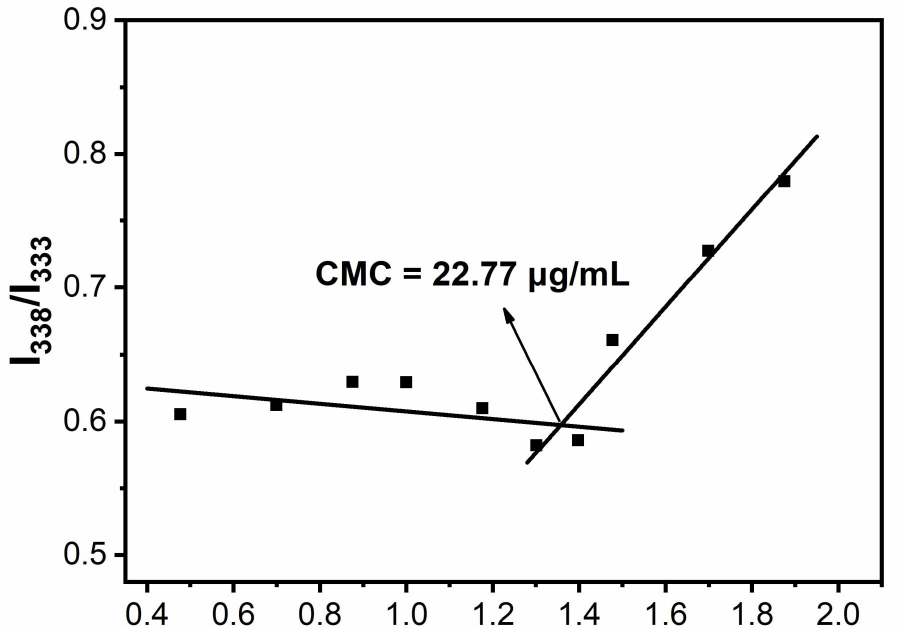

Figure S2. The critical micelle concentration (CMC) of HA@CA synthesized with feed ratio at 10%.

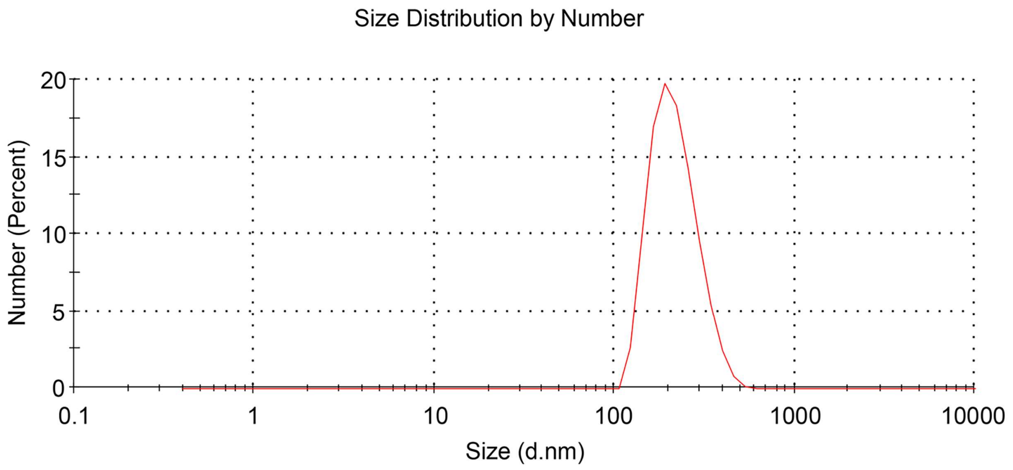

Figure S3. The hydrodynamic size distribution of HA@CAP measured by DLS.

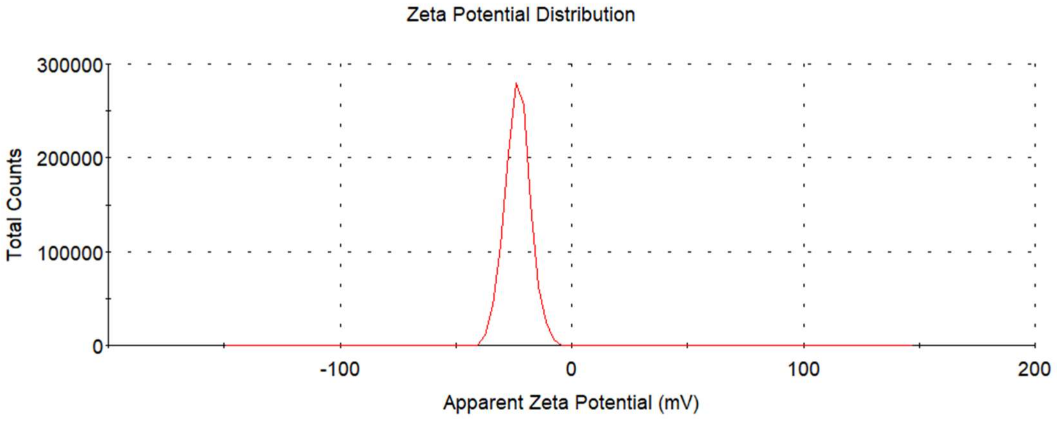

Figure S4. The zeta potentials of HA@CAP measured by DLS.

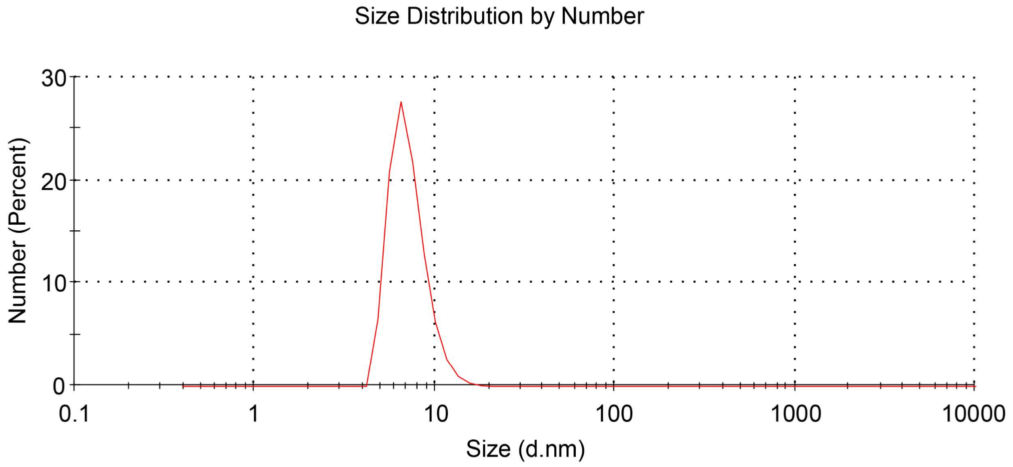

Figure S5. The hydrodynamic size distribution of completely disassembled HA@CAP in acidic conditions (pH 5.0) measured by DLS.

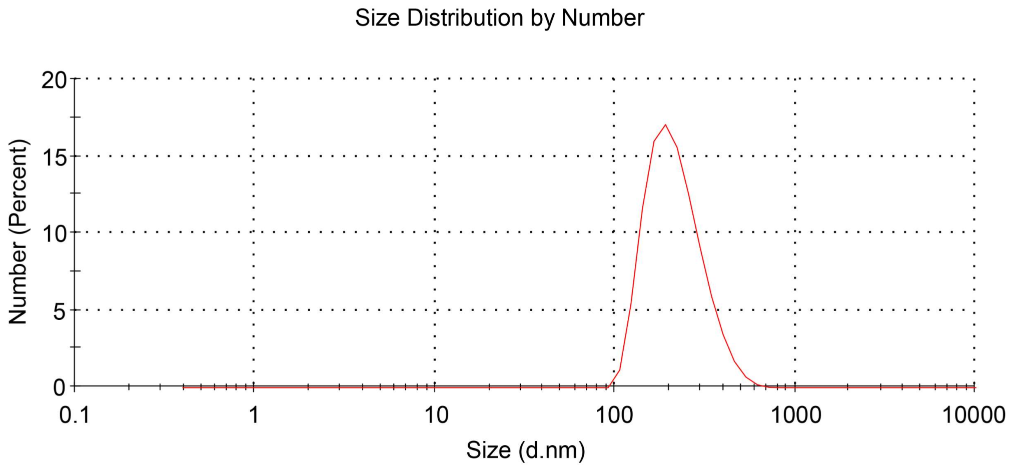

Figure S6. The hydrodynamic size distribution of HA@CAP immersed in normal physiological environment (pH 7.4) for 72 h.

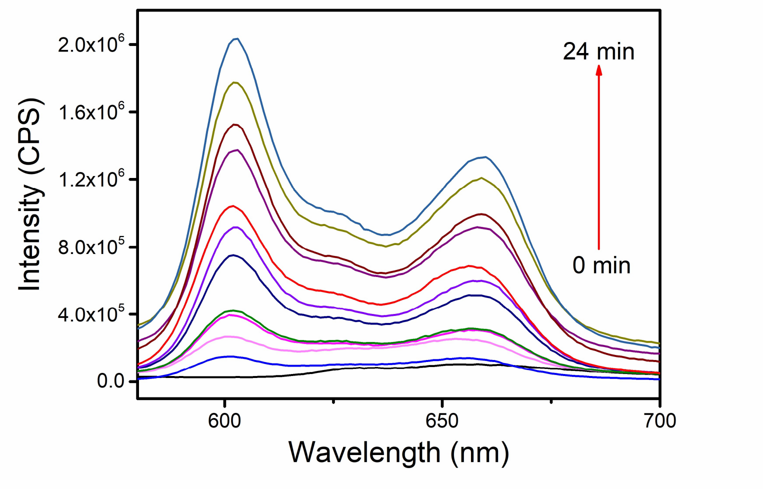

Figure S7. The fluorescence recovery phenomenon of HA@CAP after immersed in acidic environment for different times.

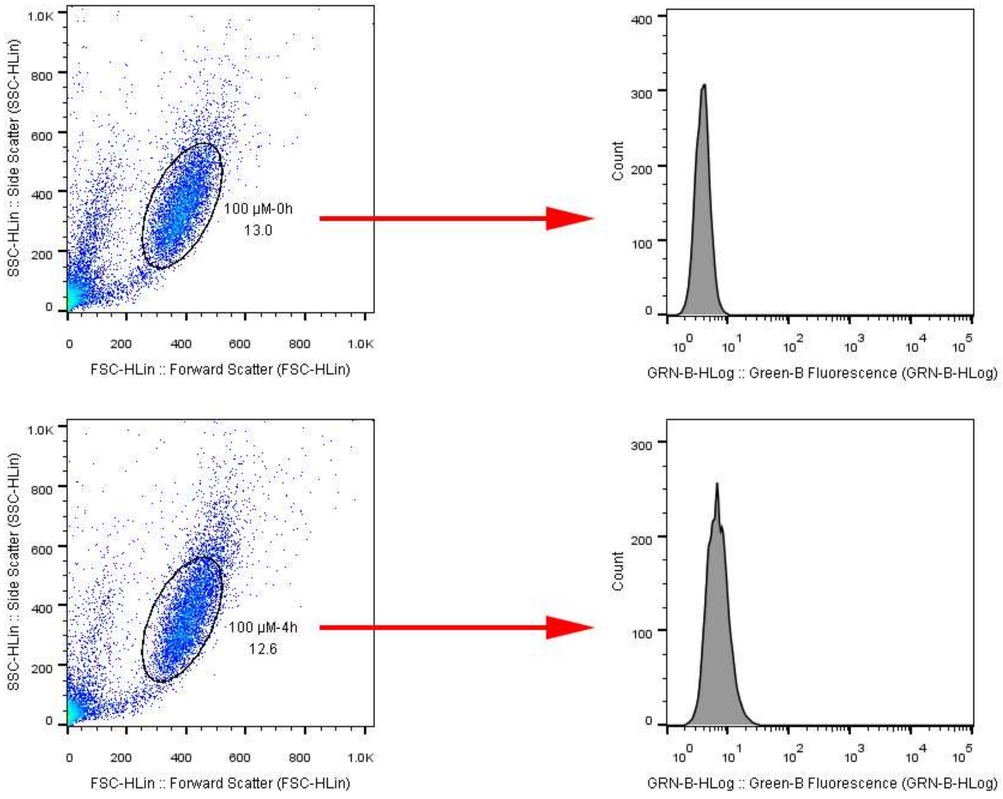

Figure S8. The representative gating strategy employed to obtain the histograms in Figure 4C.

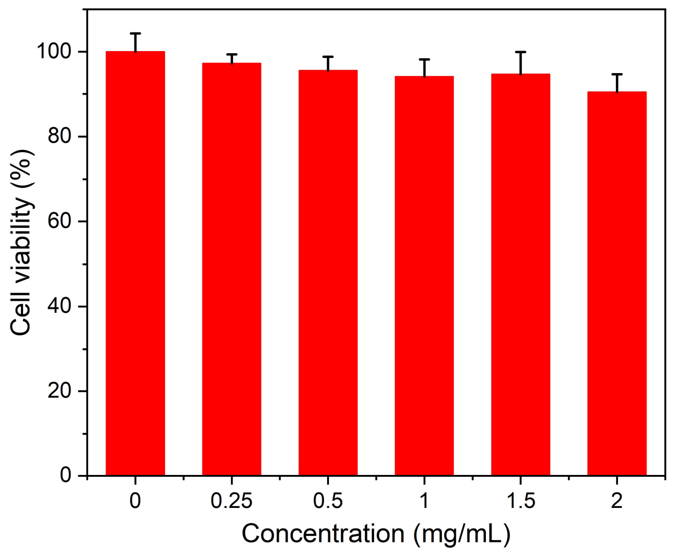

Figure S9. Cell viability of NIH-3T3 normal cells incubated with various concentrations of HA-ADH conjugates for 24 h.

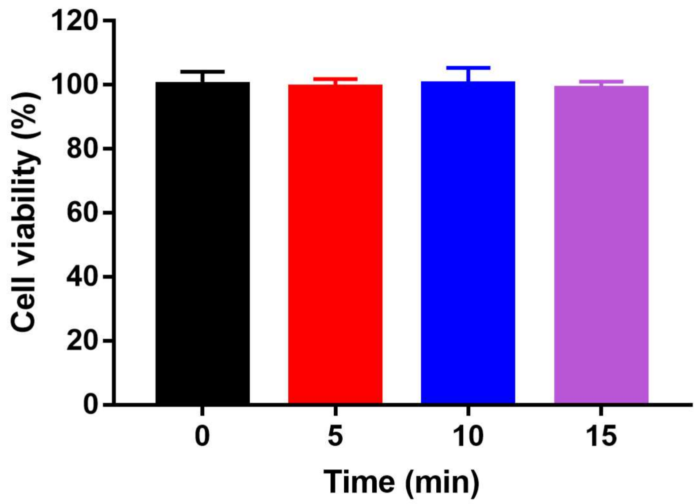

Figure S10. Cell viability of B16F10 cancer cells exposed to LED light irradiation (20 mW cm^{-2}, 630 nm) for 0, 5, 10, and 15 min after incubation for 24 h.

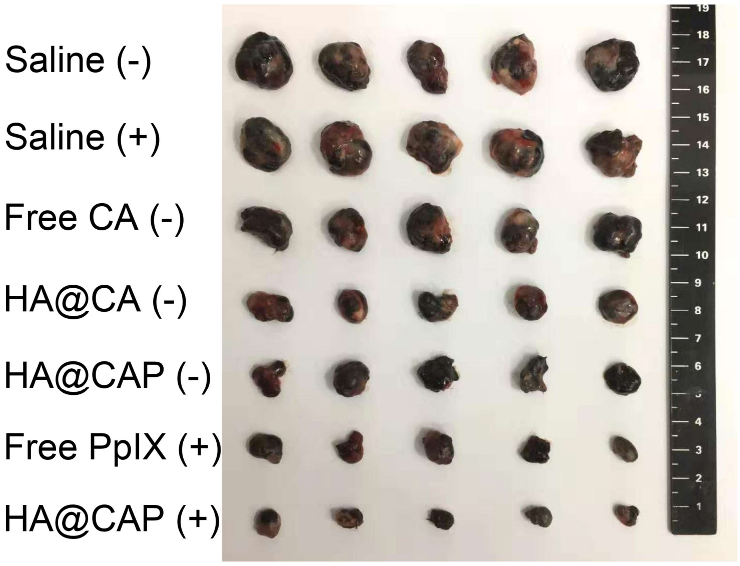

Figure S11. Representative images of excised tumor in various groups at the end of treatment.

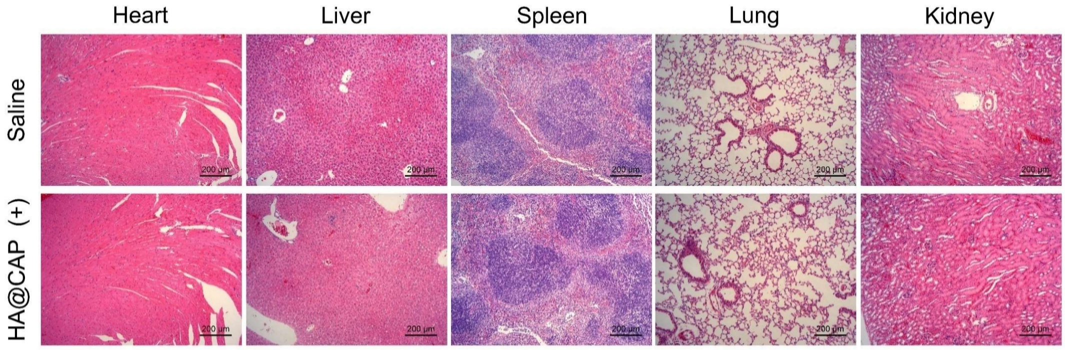

Figure S12. H&E staining of the major organs (heart, liver, spleen, lung, kidney) from saline group and HA@CAP with laser irradiation group at the end of antitumor treatment. Scale bar: 200 μm.
